# Optical Switching Using Transition from Dipolar to Charge Transfer Plasmon Modes in Ge_2_Sb_2_Te_5_ Bridged Metallodielectric Dimers

**DOI:** 10.1038/srep42807

**Published:** 2017-02-16

**Authors:** Arash Ahmadivand, Burak Gerislioglu, Raju Sinha, Mustafa Karabiyik, Nezih Pala

**Affiliations:** 1 Department of Electrical and Computer Engineering, Florida International University, 10555 W Flagler St, Miami, Florida 33174, United States

## Abstract

Capacitive coupling and direct shuttling of charges in nanoscale plasmonic components across a dielectric spacer and through a conductive junction lead to excitation of significantly different dipolar and charge transfer plasmon (CTP) resonances, respectively. Here, we demonstrate the excitation of dipolar and CTP resonant modes in metallic nanodimers bridged by phase-change material (PCM) sections, material and electrical characteristics of which can be controlled by external stimuli. Ultrafast switching (in the range of a few nanoseconds) between amorphous and crystalline phases of the PCM section (here Ge_2_Sb_2_Te_5_ (GST)) allows for designing a tunable plasmonic switch for optical communication applications with significant modulation depth (up to 88%). Judiciously selecting the geometrical parameters and taking advantage of the electrical properties of the amorphous phase of the GST section we adjusted the extinction peak of the dipolar mode at the telecommunication band (λ~1.55 μm), which is considered as the OFF state. Changing the GST phase to crystalline *via* optical heating allows for direct transfer of charges through the junction between nanodisks and formation of a distinct CTP peak at longer wavelengths (λ~1.85 μm) far from the telecommunication wavelength, which constitutes the ON state.

Localization of plasmons in metallic nanoassemblies has been analyzed by both hybridization^1^ and generalized classical multiparticle Mie theories^2^. The simplest and fundamental member of nanocomplex family is a two-member dimer with a few nanometers gap between proximal nanoparticles which is well-known for its characteristics analogous to the wave function of atomic systems^3^,^4^,^5^. Reducing the offset gap between plasmonic nanoparticles down to atomic sizes in a dimer system leads to direct (quantum) and/or indirect (Fowler-Nordheim) tunneling of optically driven charges across the subnanometer gap^6^,^7^. Excitation of an additional plasmonic mode at lower energies apart from classical plasmonic modes known as “charge transfer plasmon (CTP)” is the exotic result of this quantum mechanical process. Recently, CTPs have been induced *via* opening of a channel for direct shuttling of charges through a metallic nanowire (i.e. gold and aluminum) between neighboring plasmonic nanoparticles^8^. The conductivity and geometry of the conductive junction between proximal nanoparticles play fundamental role in tuning the position and amplitude of the induced CTPs as well as local field distribution^8^,^9^. Having direct control over the plasmonic response of nanoantennas provide unique tunability for designing new generation of nanophotonic devices^10^. In the bridged dimer limit, such an exceptional functionality can be achieved by varying the electrical or optical properties of the conductive bridge, which is not possible with conventional metallic links.

Having active control on the plasmonic properties of a given nanosystem requires presence of optoelectronically controllable components in the structure. Recently, phase-change materials (PCM) such as vanadium dioxide (VO_2_)^11^,^12^, Ge_x_Sb_y_Te_z_ (GST)^13^,^14^, and AgInSbTe^15^,^16^ are introduced as promising substances of which optical properties can be altered by electrical Joule heating or incident radiation^17^,^18^,^19^. As a specific case, Ge_2_Sb_2_Te_5_ (GST or GST225) which is formed by chalcogenide family has been utilized for nonvolatile random access memory (NVRAM) technology due to room-temperature adoptability^14^,^20^,^21^. It is demonstrated that GST can keep its state without any external energy source until the next phase change process starts^13^,^14^,^17^,^18^,^19^,^20^,^21^. The small active area filled by GST can be reversibly and quickly toggled among at least two different phases namely a conductive crystalline phase (c-GST) and highly resistive amorphous phase (a-GST) by applying either bias or incident optical pulses. Conventional plasmonic modes such as Fano resonances with tunability by optical stimuli have been observed in metal-GST-metal metamaterials^22^,^23^, and multilayer GST nanoparticles^24^.

In this article, we present a theoretical and numerical study of the plasmonic response of a metallodielectric dimer linked through a conducting bridge that can be tuned optically to be used as a platform for NIR optical switching. By inserting a short section of GST into the conducting bridge linking the nanoparticles, we show significant variations and shifts in the width and position of the induced plasmonic resonance peaks, respectively. This feature originated by the exquisite nature of GST, which reflects significant permittivity and resistivity variations due to switching between amorphous and crystalline phases when exposed to external optical stimuli. In the 0% crystallization (amorphous) phase of the PCM, the capacitive coupling between the excited modes in metallic arms results in significant dipolar peak. Adjusting the geometries of dimer system allows for tuning the extinction peaks of the dipolar resonances at the telecommunication wavelength (~1.55 μm) in this phase. Irradiating the system with a gating pulse with adequate power generates significant thermal energy resulting the phase change from amorphous to crystalline. In this limit, due to the low resistivity of the c-GST section, the induced charges can shuttle across the nanobridge forming a CTP peak and eliminating the capacitive coupling. When the GST is fully crystallized, due to significant absorption cross-section across the NIR and also transfer of charges across the dimer, the CTP mode shows substantial red-shifts (around *δ*~300 nm) to the longer wavelengths compared to the dipolar peak in the amorphous phase. Using a short light pulse, the transition from crystalline back to amorphous state can be achieved in a subnanosecond timescale^22^,^23^,^24^.

## Theoretical approach

Finite-difference time-domain (FDTD) method (Lumerical 2016) was used to study the proposed plasmonic optical switch platform. Following settings were applied for numerical analysis: The boundaries were surrounded by highly absorptive perfectly matched layers (PMLs) and the incident beam for crystallization was a broadband plane wave with the bandwidth of 400 nm–1600 nm, with the irradiation power of *P*_*0*_ = 3.2 μW, beam fluence of 60 Jm^−2^, pulse duration of 2.7 ns, and repetition of 10 KHz (Fig. S1a and b). The distance from source to the targeted nanostructure was set to 1 μm. We also defined a light source with the duration of 0.9 ns and irradiation power of 5.5 mW to provide the required energy for the change from crystalline to amorphous phase (amorphization process). For more details about the thermal heating see the Supplementary Information file. To provide accurate results, the workplace discretization was set to 0.5 nm in all of the axes. According to the Courant stability, the simulation time step is set to *dt* ~0.1 fs. The permittivity of the glass (SiO_2_) substrate was set to 2.1 according to the Palik constants^25^, while the dielectric function empirically determined by Johnson-Christy was used for the gold plasmonic elements^26^. The corresponding complex permittivity for different phases of the GST were taken from the experimental data reported by Shportko *et al*.^27^.

**Figure 1:**
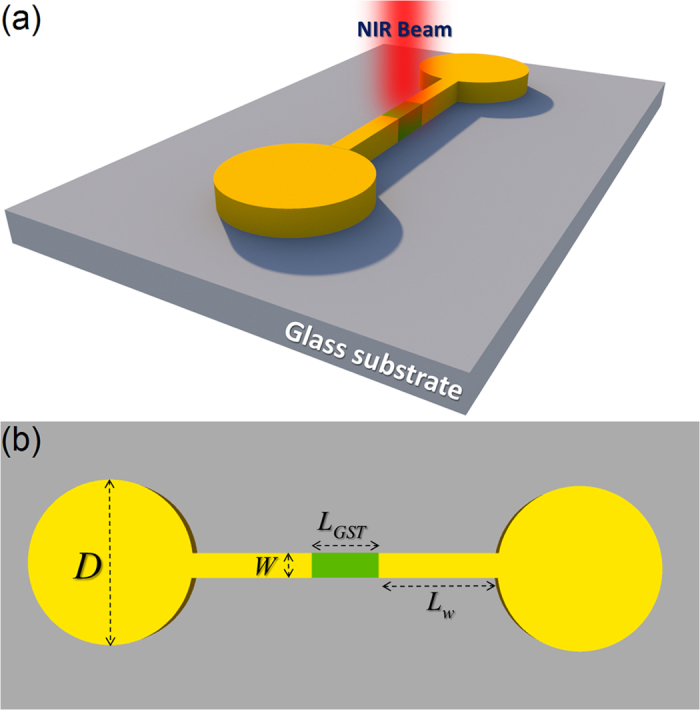
(**a**) An artistic rendering of the proposed tunable dimer nanoantenna on a glass substrate. (**b**) A top-view plot of the dimer nanoantenna with corresponding geometrical parameters.

Figure 1a shows an artistic rendering of the proposed nanosystem composed of a couple of plasmonic nanoparticles linked to each other by a metallic nanowire bridge and a GST section located in the middle of the bridge. The intermediate GST layer is in perfect contact with the metallic bridge in both sides in the numerical analysis. The geometrical parameters for each part of the dimer nanoantenna are indicated in Fig. 1b. Since our goal is to show the effect of the GST layer on the excited plasmonic modes, the diameter of the satellite metallic disks are be fixed to *D* = 200 nm, and the length of the metallic parts (*L*_*M*_) in the bridge design is varied depending on the size of the GST section. The thickness of the entire system is fixed to 45 nm. Excitation of the CTP mode strongly depends on the geometry and conductivity of the junction between plasmonic elements, which allows for transition of charges swiftly. Thus, having active control on the optical properties of this nanobridge would be useful to control the excited CTP mode effectively. This tunability is provided by applying conductivity variations *via* phase switching in the GST section between the junctions. The wavelength-dependent conductivity of the GST layer can be defined as^8^,^28^:


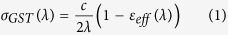


where, *c* is the velocity of light in a vacuum, *ε*_*eff*_ (*λ*) is the effective permittivity of GST bridge in the intermediate phases that can be defined by using Lorentz-Lorenz effective-medium expression at crystallization level^29^,^30^,^31^:


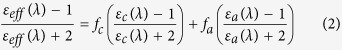


where *f*_*i*_ is the volume function of the *i*th phase as follows:


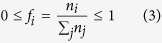


where *n*_*j*_ is the density of the *j*th phase. Transition (switching) between amorphous and crystalline phases is accomplished by formation of localized Joule heating due to illumination with specific power for an adequate duration. The reported critical temperature for this phase change is ~433 K^32^,^33^, which must be achieved *via* light-matter interaction by tuning the incident light intensity and absorption coefficient (*Q*_*abs*_). It is shown that in the quasi-electrostatic limit, the photothermal heat process for compositional nanostructures can be characterized by using multicapacitive cascading approach^34^,^35^,^36^. This theoretical model contains the enhanced E-field corresponding with the individual parts of a plasmonic nanostructure. The absorbed photothermal heat energy (*E*_*H*_) in the metallodielectric system can be determined by^37^,^38^: *E*_*H*_ = *AQ*_*abs*_*F*(*r)*, where *A* is the entire area of the plasmonic dimer, and *F*(*r)* represents the optical fluence of the incident gating pulse (see the Supplementary Information). Using the settings for the Gaussian beam source that are explained in the numerical methods, and also by tuning the dimer geometries, the required thermal heat is produced to switch the GST phase. It should be underlined that a-GST shows low-loss behavior at lower energies (below *E* < 0.8 eV) which gives rise to drastically poor absorption coefficient^39^ while the c-GST shows significant absorption cross-section across this spectra. The absorption cross-section for all of the examined regimes are calculated and demonstrated in the Supplementary Information, which are consistent with the calculated extinction profiles.

## Analyzing the proposed metallodielectric bridged dimer

**Figure 2:**
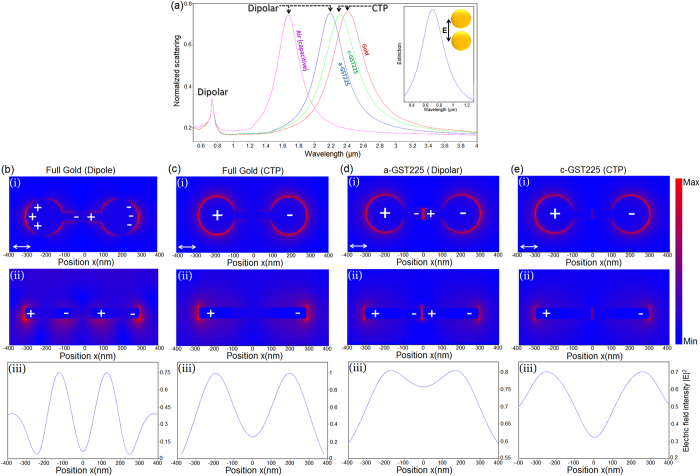
(**a**) Normalized extinction spectra of the bridged dimer in GST-mediated, full-metallic, and air regimes. The inset is the normalized extinction cross-section for the same dimer without conductive junction and plotted for the capacitive coupling regime (the offset gap between nanoparticles is 15 nm). (**b**,**c**) E-field maps across the bridged dimer for dipolar and CTP resonant mode in full metallic regime, respectively, for both (i) top-view and (ii) cross-sectional views. (**d**,**e**) The local E-field distributions correlating with the dipolar and CTP resonant peaks, respectively, for both (i) top-view and (ii) cross-sectional views. (iii) The electric-field intensity diagrams |*E*|^2^ for the metallodielectric and metallic dimers at the position of CTP and dipolar modes.

Figure 2a shows the normalized extinction spectra for the dimer system for four different compositional regimes of the bridge. We consider a bridge with the total length of *L*_*B*_* = *100 nm with a GST section with the length of *L*_*GST*_ = 10 nm and assume that the GST is in amorphous state initially with 0% crystallization and then switched to full crystallization state (100%). For the entirely gold bridge without PCM, a classical bright dipolar resonant mode is induced due to capacitive coupling between nanoparticles at *λ* = 0.73 μm, and a pronounced CTP extreme appeared at the longer wavelengths (*λ* = 2.4 μm) due to the shuttling of the charges across the dimer through the conductive pathway. By removing a small part of the metallic wire and making an offset gap of 10 nm (air space) as a capacitive region at the center of the metallic bridge, the same dipolar mode is observed due to coupling of the distant nanodisks, while a significant dipolar peak is also induced due to capacitive coupling between rectangular bridges. It should be noted that the second extreme is more intense due to small gap distance between bridge arms. Insertion of a short a-GST section with high resistivity (in the range of ~10^4 ^Ω.cm) at the center of the bridge causes the more intense dipolar peak to red-shift to the longer wavelengths (*λ* = 2.2 μm). Due to the low-loss behavior of the a-GST at low energies (*k*~0), the extinction coefficient at the GST interface is negligible, hence, the induced dipolar mode remains intense.

Switching the state of the GST to full crystallized phase (100% crystallization) results in a substantial decrease in the corresponding resistivity (~10^−3 ^Ω.cm)) and charge transfer becomes dominant mechanism (negligible capacitive coupling). The result of such a charge transfer is formation of a significant CTP peak at *λ* = 2.3 μm. Here, the notable red-shift in the position of CTP mode is caused by the absorptive behavior of the c-GST layer at low energies. As shown in the figure, there is *δ*~100 nm difference between dipolar and CTP modes for different GST phases. Position and amplitude of the leftmost dipolar mode shoulder is particularly independent of the conductive bridge properties for all examined regimes. The inset is the comparative extinction curve for an isolated dimer structure with capacitive coupling showing the induced dipolar peak around 0.7 μm. Figure 2b and c, and subsequent diagrams exhibit the E-field maps (i and ii) and intensity (iii) for the position of dipolar and CTP modes in full gold regime, respectively, revealing the charge distribution difference with the CTP mode. For the presence of GST intermediate layer, the gap distance between nanodisks is set to 15 nm and the corresponding local electric-field (E-field) distribution across the antenna for three-different regimes are shown in Fig. 2d and e. For the junction between plasmonic nanodisks with a-GST, opposite charges are concentrated in the nanodisks as well as around the dielectric junction (Fig. 2d), while a concentration of E-fields is visible across the GST section caused by its resistive behavior, which causes a small damping in the induced dipolar peak. For the c-GST, the E-field is much lower, and the extinction of plasmons is still obvious (Fig. 2e), however, the charges can pass through the bridge and the capacitive coupling is eliminated. In contrast, in the entirely gold bridge regime, the charges are transported easily and their concentration around and in the middle of the bridge is invisible compared to the other metallodielectric regimes (Fig. 2c). Comparing the calculated E-field intensity profiles for all of the examined regimes (Fig. 2(iii)), with the presence of the dielectric material (GST), the field intensity at the central part of the nanoantenna is reduced, which is consistent with the numerical charge distribution maps.

**Figure 3:**
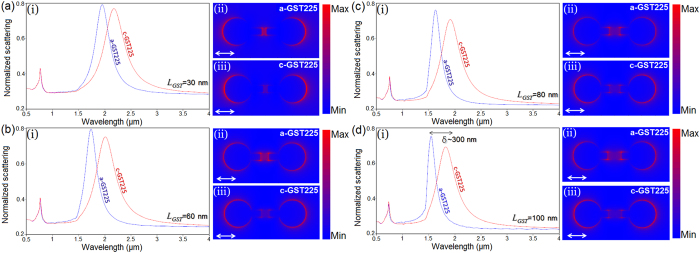
(**a**–**d**) (i) Normalized extinction spectra for the GST-mediated bridged dimer, while the *L*_*GST*_ is variant. (ii) and (iii) The corresponding E-field maps for *L*_*GST*_ variations for both amorphous and crystalline phases.

In continue, we optimize the spectral tunability of the proposed metallodielectric dimer by varying its geometry and monitoring the dipolar and CTP mode response. For the full metallic bridge, it was shown that increasing the length of the nanojunction leads to longer time and lengthier path for travelling of the induced charges across the junction, resulting reduction in the quality of the CTPs significantly^8^,^9^. In the presented case, different electrical and optical properties of the amorphous and crystalline phases of GST provides the key advantage for tunability^20^,^21^. The resistance contrast for two opposite states of the GST (*R*_*a*_/*R*_*c*_) is around ~10^7 ^39,40,41. By keeping the overall length of the interconnecting nanobridge fixed at *L*_*B*_ = 200 nm, and by varying only the length of the GST junction, we shifted the resonant mode to the shorter spectra for both a-GST and c-GST as shown in Fig. 3. By increasing *L*_*GST*_ (10 nm ≤ *L*_*GST*_ ≤ 100 nm) and reducing the length of the metallic bridges (the entire bridge length is kept fixed), for the a-GST phase, the dipolar peak is blue-shifted to the shorter wavelengths and became narrower including a small damping in the amplitude of the peak. For the c-GST phase, the CTP extreme is red-shifted for all of the examined sizes, while this shift is approximately three-times larger than the previous analysis (*δ*~300 nm). For instance, for the longest GST length (*L*_*GST*_ = 100 nm), a narrow dipolar resonant peak is induced around *λ*~1.54 μm for amorphous state, while for the same geometry and crystallized regime the resonant mode is shifted to *λ*~1.85 μm (as a CTP peak) which is useful for designing NIR optical telecommunication devices^42^,^43^,^44^,^45^. The E-field maps next to the normalized extinction profiles (Fig. 3(ii) and (iii)) provide a better view of the effect of the GST length variations on the CTP.

**Figure 4:**
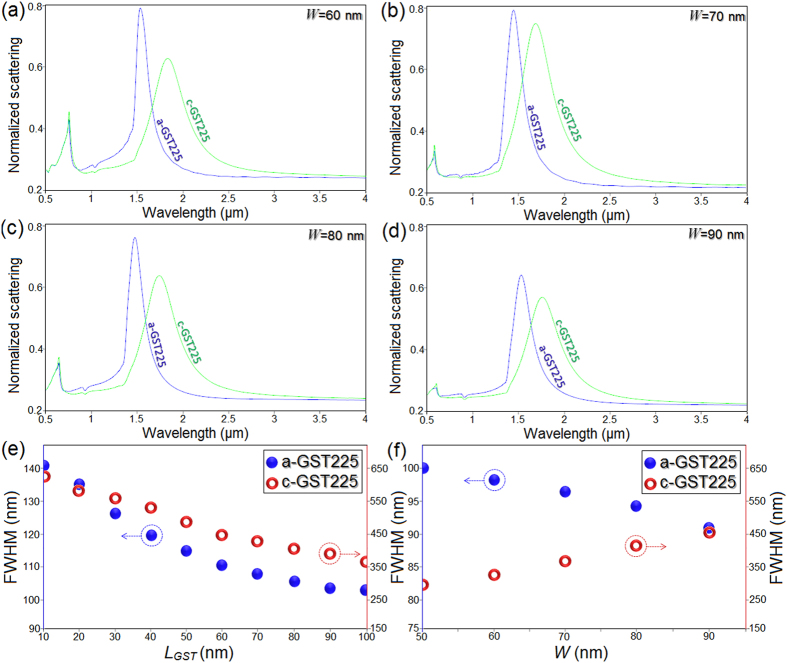
(**a**–**d**) Normalized extinction profiles for the GST-mediated bridge dimer antenna with different bridge thickness (*W*). (**e**,**f**) The FWHM profiles as a function of *L*_*GST*_ and *W* for the presence of both a-GST and c-GST layers at the center of bridge.

Next, we analyze the effect of further geometrical variations on the resonant extinction peaks as demonstrated in Fig. 4a–d. We increased the width of the nanobridge as *W* = 60 nm, 70 nm, 80 nm, and 90 nm for both phases of the GST section. For *W* = 60 nm, the resonant dipolar and CTP peaks in both states are blue-shifted slightly (Fig. 4a), while by increasing the width of the bridge, both peaks show similar shift to the higher energies with the amplitude comparable with earlier investigations (Fig. 4b and c). Here, increasing the widths of the bridge up to *W* = 80 nm leads to blue-shift of both resonance peaks to the higher energies. However, continuous increase in the width of the GST and extinction of gold parts of the junction also causes significant damping (decoupling) in the peaks. This decay in the amplitude and energy of CTP mode (for c-GST) is significant in Fig. 4d. The blue-shift and amplitude damping in both dipolar and CTP modes can be described based on the behavior of the GST material. For the presence of a-GST at the junction, the excited modes in individual metallic arms can couple efficiently leading to formation of strong dipolar mode in the range of 60 nm < *W* < 80 nm. In this regime, the dipolar peak slightly blue-shifts to the higher energies. However, by increasing the width of the bridge for *W* > 90 nm, the energy losses increases drastically leading to an appreciable damping in the amplitude of the dipolar peak. For the presence of c-GST, the charges can transit across the bridge and by increasing the width of the bridge, therefore, more charges can travel to the outermost nanodisks and gives rise to blue-shift in the position of the induced CTP. However, for *W* > 90 nm, due to inherent lossy behavior of metallic components and also absorptive behavior of crystalline PCM for *E* > 0.8 eV (see Fig. S2), the peak is damped drastically. This decay in both dipolar and CTP is accompanied with a noticeable and progressive damping in the leftmost dipolar shoulder including a giant blue-shift in the position of the dipolar shoulder. Figure 4e and f compare the corresponding full wave at half maximum (FWHM) of the induced dipolar and CTP extremes for different phases as a function of GST length and widths. By increasing the length of the GST section, the corresponding FWHM is reduced substantially for both phases of PCM, showing the required narrowness for accurate and fast operations (Fig. 4e). For the entire bridge width variations, the corresponding FWHM increased (decreased) with the increasing width for c-GST (a-GST) state. These opposing trends can be attributed to the different loss mechanism of the dipolar and CTP modes as explained above. Possessing sharp and narrow peak with small FWHM would help to design high-precision plasmonic devices^45^,^46^,^47^,^48^,^49^,^50^. However, the obtained narrowness for the wider bridge is accompanied with dramatic damping in the induced dipolar and CTP extremes.

Using the GST material in the geometry of a nanostructure needs for a capping layer to prevent its degradation during phase transition at high temperatures and also at high number of operation cycles^51^. We analyzed the effect of the presence of capping layer (SiO_2_) with the thickness of 30 nm in the Supplementary Information (Fig. S3). It is shown that the presence of capping layer does not change the extinction cross-section significantly, due to its negligible effect on the effective refractive index of the entire structure. In other words, the extinction coefficient (*k*) of the overall bridged dimer does not change significantly, therefore, we can be sure that presence of such a dielectric layer does not affect the performance of the proposed nanoswitch intensely. The other critical issue here is the crystallization level of the GST material in the geometry of the bridged dimer. Considering the fact that reaching full crystallization level (100%) in experiments is challenging, we analyzed the spectral response of the structure for the lower crystallization level (95%). The results and explanations for these analysis are presented in Fig. S4.

## Optically tunable NIR switch

**Figure 5:**
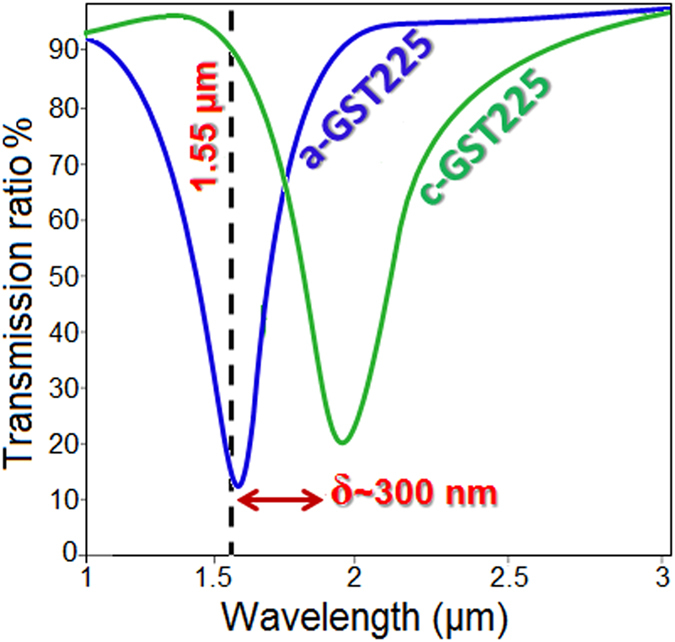
The transmission ratio of the GST-mediated metallodielectric switch in OFF (amorphous) and ON (crystalline) states.

For conventional all-optical and electro-optical plasmonic switches that are tailored to operate at the NIR such as metamaterials^52^,^53^,^54^, and waveguides^55^,^56^,^57^,^58^, low cross-talk and field leakage, and fast switching are the fundamental requirements of high performance. Sharpness and position of the induced dipolar and CTP modes play key roles in determining the suitability of the proposed nanoplatform for optical switching. Our foregoing analysis show that the nanostructure with the geometry of *L*_*GST*_ = 100 nm, *W* = 50 nm, *T* = 45 nm, and *D* = 200 nm is the best candidate for switching applications. Providing approximately *δ*~300 nm shift for the resonance peak between two different phases (Fig. 3d) and fast switching from amorphous to the full crystallization state (requiring a few nanoseconds) as well as switching back to amorphization (requiring hundreds of femtoseconds)^45^,^49^,^59^, these structures could be used for designing fast and efficient plasmonic photonic switches. We demonstrate the switching performance of the studied metallodielectric nanostructure by adjusting the position of the GST-mediated resonant peak centered at 1.55 μm and analyzing the transmission ratio, as shown in Fig. 5. First we assume that the GST section of the bridge is in amorphous phase (OFF state of the switch). Then, by applying a high-power gating pulse signal^45^,^49^, crystallization process is started and the resonance wavelength shifts to the lower energies (ON state of the switch). As it was mentioned earlier, in order to reverse the switching process from ON to OFF state, a gating beam with higher power and shorter duration must be applied (see the Supplementary Information). According to the transmission ratio profile, the modulation depth across the telecommunication band (*λ* = 1.55 μm) is around 88%. As a specific case, for *λ* = 1.55 μm, the corresponding insertion loss (*IL*) for switching from OFF to ON state is calculated based on the monitored power (*P*_*m*_) and the incident (*P*_*i*_) by: *IL* = −10Log_10_ (*P*_*i*_/*P*_*m*_)^60^,^61^, which is yields ~4.5 dB for *L*_*GST*_ = 100 nm.

## Conclusions

To conclude, we have proposed and analyzed a platform of integrated GST-mediated bridged plasmonic dimer for fast, nonvolatile, all-optical switching applications operating at the telecommunication band. We have shown that the GST section in amorphous phase hinders direct transfer of charges across the nanobridge and acts as a capacitive region resulting a distinguished dipolar extinction peak at the global telecom wavelength (1.55 μm), which constitutes the OFF state of the switch. By applying a high power gating pulse to produce the required photothermal heat energy, a-GST switches to the crystalline orientation (c-GST) and attains low resistivity at the operating domain, leading to a CTP peak at the lower energies (*δ*~300 nm apart from the dipolar peak for the a-GST), corresponding to the ON state. Fast and reversible switching performance of the analyzed metallodielectric nanostructure could be used for designing efficient all-optical and optoelectronic devices for telecommunication applications.

## Additional Information

**How to cite this article**: Ahmadivand, A. *et al*. Optical Switching Using Transition from Dipolar to Charge Transfer Plasmon Modes in Ge_2_Sb_2_Te_5_ Bridged Metallodielectric Dimers. *Sci. Rep.* **7**, 42807; doi: 10.1038/srep42807 (2017).

**Publisher's note:** Springer Nature remains neutral with regard to jurisdictional claims in published maps and institutional affiliations.

## Supplementary Material

Supplementary Information
