# Supplementary material for: Optical Switching Using Transition from Dipolar to Charge Transfer Plasmon Modes in Ge2Sb2Te5 Bridged Metallodielectric Dimers
Source: Sci Rep. 2017 Feb 16;7:42807. doi: 10.1038/srep42807 (PMC5311934; doi:10.1038/srep42807)
Supplement: Supplementary Information [file srep42807-s1.doc]

Supporting Information:

Optical Switching Using Transition from Dipolar to Charge Transfer Plasmon modes in Ge_2_Sb_2_Te_5_ Bridged Metallodielectric Dimers

*Arash Ahmadivand,*†* *Burak Gerislioglu,*† *Raju Sinha, Mustafa Karabiyik, and Nezih Pala*

*Department of Electrical and Computer Engineering, Florida International University, 10555 W Flagler St., Miami, Florida 33174, USA*

*Corresponding Author: [*a*](mailto:a_ahmadivand@iau-ahar.ac.ir)*ahmadiv@fiu.edu*

†Equal contribution.

**Contents**

**1**. Source setting and heat calculations.

**2**. Absorption cross-section for metallic and metallodielectric bridged dimer.

**3.** The effect of capping SiO_2_ coverage on GST layer.

**4**. The effect of low-crystallization percentage on the plasmonic response.

**Figures**

**Figure S1.** Normalized optical signal amplitude as a function of time delay.

**Figure S2.** Normalized absorption spectra for a bridged dimer system in three different regimes.

**Figure S3.** Normalized extinction spectra for a bridged dimer system for the presence of SiO_2_ capping layer.

**Figure S4.** Normalized extinction spectra for a bridged dimer system for different crystallization percentages.

**1. Source setting and heat calculations.**

To excite surface plasmon resonances and changing the GST phase between amorphous and crystalline states, we used two Gaussian beam sources with the specified settings that are explained in the theoretical description in the main article. Here, we describe the methodology for produced heat in the antenna *via* light-mater interaction. To change the amorphous phase of the intermediate-GST layer to the crystalline state, we used an extremely short pulse signal with the duration of 2.65 fs, as shown in Figure S1a. Using a general theory, it is shown that the temperature variations in a specific surface due to illumination of Gaussian beam is given by heat conduction theory for surface heat source:^1^

(1)

where

(2)

where *q_0_* is the flux density of the heat source, *κ* is the thermal conductivity, *v* is the volume of the object, *k_D_* is the thermal diffusivity, *r* is the corresponding radial distance defined parallel to the surface of the illumination are from the origin of the radiation, *z* is the vertical distance estimated by computing the perpendicular to the surface from the source, and *φ* is the polarization angle of the incident radiation. Such a complex calculation can be simplified using the proposed method by Chen *et al*.^2^ for a Gaussian beam source by considering the fluence of the incident light over a given structure as a function of distance from the source to the sample (*r*):


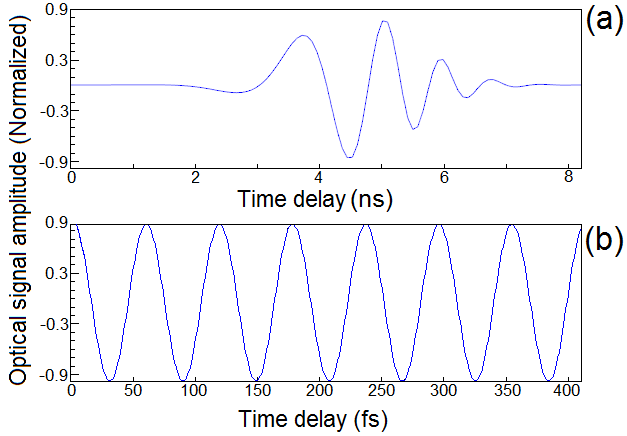


**Figure S1**. (a) and (b) Normalized optical signal amplitude as a function of time delay for the pulse and continuous wave sources, respectively.

$F(r)=\frac{2P_{0}e^{-\frac{2r^{2}}{w^{2}}}}{\pi w^{2}f_{r}}$ (3)

where *P_0_* is the incident power from source (3.2 μW), *w* is the Gaussian beam waist (set to 600 nm), and *f_r_* is the repetition rate of the incident pulse (10 KHz). As a result, the produced heat power can be easily defined by:^2,3^

(4)

where *Q_abs_* is the numerically absorption coefficient, *ϕ* represents the optical fluence of the incident beam, *τ* is defines the time constant of the incident pulse in the range of a few nanosecond, and *t_0_* is the time delay of the pulse peak. Following settings are applied for our analysis: *ϕ*=60 Jm^-2^, *τ*=2.2 ns, and *t_0_*=3.5 ns. In contrast, for amorphization process, as shown in Figure S1b, we particularly used continues wave steering in the metallodielectric dimer with shorter exposure time and higher power (5.5 mW) than the crystallization process for shorter duration (~0.9 ns).^4,5^

**2. Absorption cross-section for metallic and metallodielectric bridged dimer.**

The normalized absorption cross-sections for the both metallodielectric and metallic bridged dimer with the finally reported geometries as: *D*=200 nm, *L_GST_*=100 nm, *T*=45 nm, and *W*=50 nm are illustrated in Figure S2. Obviously, the obtained profiles are consistent with the extinction spectra, and the maximum absorption is monitored at the position of dipolar and CTP modes for all of the examined regimes for the junction nanowire. In addition, a weak absorption shoulder is emerged at the dipolar mode wavelength. For the GST-mediated metallodielectric regime, the absorption amplitude with the presence of c-GST is more than the a-GST layer due to its lossy behavior across the NIR (Figures S2a and S2b). For the bridged dimer with full metallic junction, the absorption spectra is intensified, while the peak is still remained distant from the telecommunication band, showing the exquisite feature of GST-mediated nanostructure. In this regime, the superior absorption of the metallic dimer is obvious.


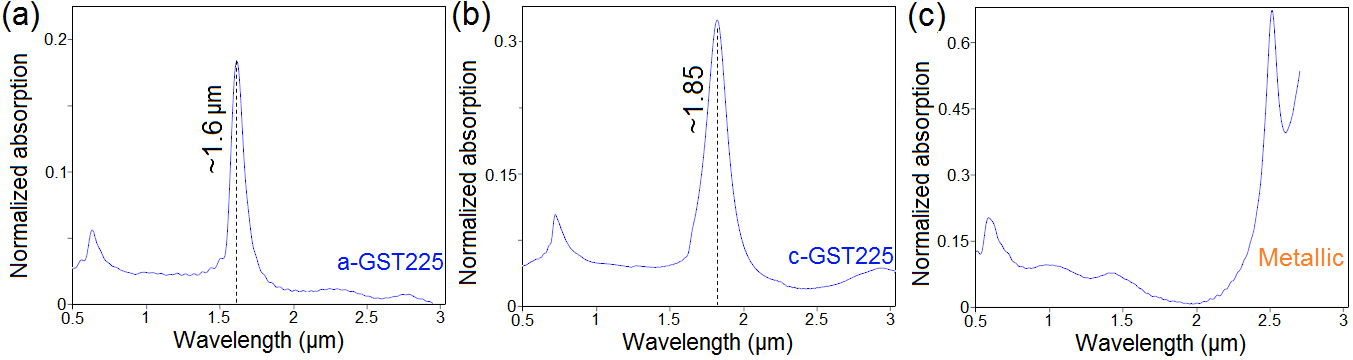


**Figure S2**. Normalized absorption spectra for a bridged dimer system with the (a) a-GST-mediated, (b) c-GST-mediated, and (c) fully metallic junction.

**3. The effect of capping SiO_2_ coverage on GST layer.**

Using the GST material in the geometry of a nanostructure needs for a capping layer to prevent its degradation during phase transition at high temperatures and also at high number of operation cycles. However, using such a cap layer to keep the GST intact for a number of applications can be neglected for the applications that rarely needs for switching between phases.^6^ For the examined case of nanoplatform for switching purposes, large numbers of switching between intermediated GST layer phases is required. Here to show the influence of capping layer, SiO_2_ and Si_3_N_4_ layers can be employed. As an example we plotted the corresponding normalized extinction cross-section in Fig. S3 for the cases of with and without a SiO_2_ capping layer. The thickness of SiO2 is set to 30 nm. Obviously, the presence of capping layer does not change the extinction spectra significantly, due to its negligible effect on the effective refractive index of the entire structure. In other words, the extinction coefficient (*k*) of the overall bridged dimer does not change significantly, therefore, we can make sure that presence of such a dielectric layer does not affect the performance of the proposed nanoswitch appreciably.


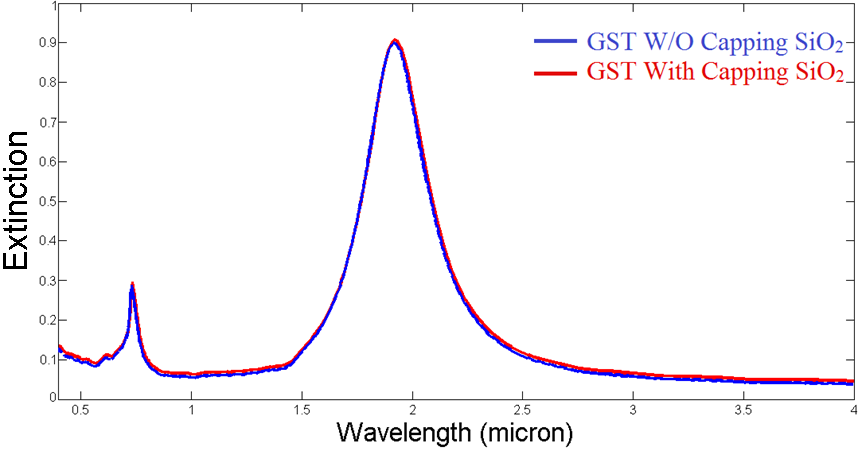


**Figure S3**. Normalized extinction spectra for a bridged dimer system with the presence of capping SiO_2_ layer as a coverage for the GST layer.

**4. The effect of low-crystallization percentage on the plasmonic response.**

Here, we initially used the proposed experimental data in the literature and recent analysis for the amorphous (0% crystalline) and crystalline (100% crystalline) states of GST layer. It should be noted that for lower crystallization regime, however, the GST layer is more conductive than purely amorphous case, therefore, we expect formation of the same CTP peak with subtle variations in the position (blue-shift to the shorter spectra). To show such an effect, we compared and demonstrated the extinction spectra for a GST-mediated bridged dimer structure with partially crystallized (95%) and fully crystallized (100%) states in Fig. S4. Obviously, for lower crystallization of phase-change material, a subtle blue-shift in the position of CTP extreme is observed due to variations in the conductivity of the entire bridge. This shift in the resonant mode position is due to direct relation between the crystallization percentage and AC conductivity.


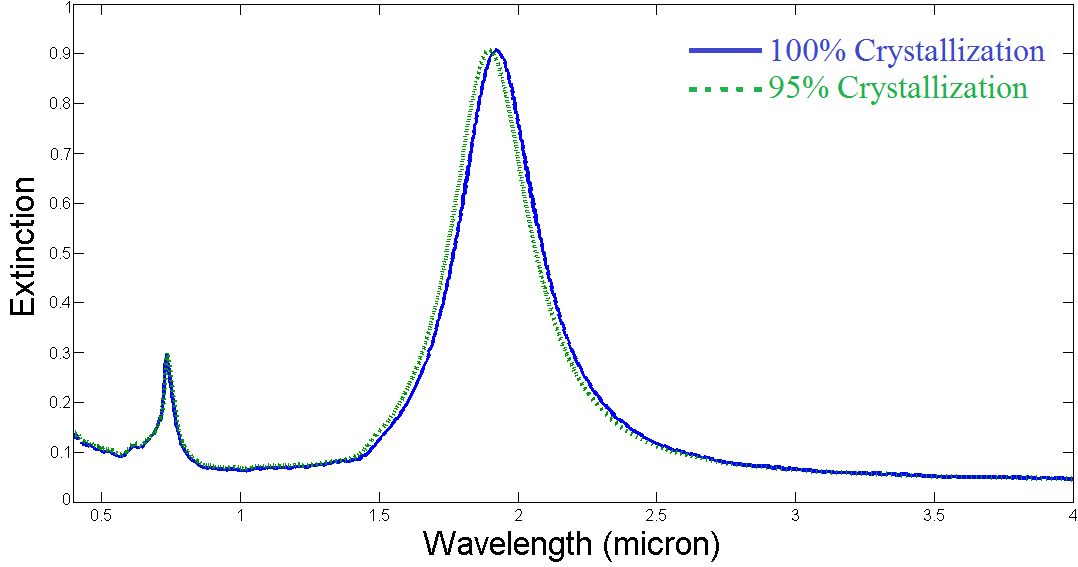


**Figure S4**. Normalized extinction spectra for a bridged dimer system for different crystallization percentage of GST layer.

**References**

1. Arata, Y., & Miyamoto, I. Some fundamental properties of high power laser beam as a heat source (Report 3). *Trans. Jpn. Weld. Soc.* **1972**, 3, 163-180.
2. Chen, X., Chen, Y., Yan, M., & Qiu, M. Nanosecond photothermal effects in plasmonic nanostructures. *ACS Nano* **2012**, 6, 2550-2557.
3. Baffou, G., & Quidant R. Thermo-plasmonics: using metallic nanostructures as nano-sources of heat. *Laser Photonics Rev*. **2013**, 7, 171-187.
4. Li, P., Yang, X., Mab, T. W. W., Hanss, J., Lewin, M., Michel, A. –K. U., Wuttig, M., & Taubner, T. Reversible optical switching of highly confined phonon-polaritons with an ultrathin phase-change material. *Nat. Mater*. **2016**, 15, 870-876.
5. Min, G., & Veronis, G. Absorption switches in metal-dielectric-metal plasmonic waveguides. *Opt. Express* **2009**, 17, 10757-10766.
6. Bakan, G., Gerislioglu, B., Dirisaglik, F., Jurado, Z., Sullivan, L., Dana, A., Lam, C., Gokirmak, A., & Silva, H. Extracting the temperature distribution on a phase-change memory cell during crystallization. *J. Appl. Phys*. **2016**, 120, 164504.
